# A New Coarse-Grained Model for *E. coli* Cytoplasm: Accurate Calculation of the Diffusion Coefficient of Proteins and Observation of Anomalous Diffusion

**DOI:** 10.1371/journal.pone.0106466

**Published:** 2014-09-02

**Authors:** Sabeeha Hasnain, Christopher L. McClendon, Monica T. Hsu, Matthew P. Jacobson, Pradipta Bandyopadhyay

**Affiliations:** 1 School of Computational and Integrative Sciences, Jawaharlal Nehru University, New Delhi, India; 2 Skaggs School of Pharmacy and Pharmaceutical Sciences, University of California San Diego, San Diego, California, United States of America; 3 Graduate Group in Biophysics, University of California San Francisco, San Francisco, California, United States of America; 4 Department of Pharmaceutical Chemistry, University of California San Francisco, San Francisco, California, United States of America; Jacobs University Bremen, Germany

## Abstract

A new coarse-grained model of the *E*. *coli* cytoplasm is developed by describing the proteins of the cytoplasm as flexible units consisting of one or more spheres that follow Brownian dynamics (BD), with hydrodynamic interactions (HI) accounted for by a mean-field approach. Extensive BD simulations were performed to calculate the diffusion coefficients of three different proteins in the cellular environment. The results are in close agreement with experimental or previously simulated values, where available. Control simulations without HI showed that use of HI is essential to obtain accurate diffusion coefficients. Anomalous diffusion inside the crowded cellular medium was investigated with Fractional Brownian motion analysis, and found to be present in this model. By running a series of control simulations in which various forces were removed systematically, it was found that repulsive interactions (volume exclusion) are the main cause for anomalous diffusion, with a secondary contribution from HI.

## Introduction

As computer simulations of biomolecules advance, efforts are underway to mimic the behavior of many macromolecules at the same time. Modeling a cell using physics-based techniques is one of the ambitious goals in understanding behavior of many macromolecules. Among the different classes of models to describe a cell, one class treats macromolecules as particles without any volume and solves either a stochastic equation of motion or models a cell as a reaction-diffusion system [1]–[5]. In the other class of model, researchers have taken initial steps towards building three-dimensional virtual cells with a molecular perspective although often the resolution of the macromolecules is much coarser than the molecular resolution. These important steps and complementary modeling with different resolution have the common goal of investigating cellular processes at larger length scales and longer time scales and predicting how a cell might respond to any number of perturbations such as drugs, diet, mutations, etc. [6]–[13].

Here, we describe the cytoplasm of one of the most commonly studied bacterial cells, *E*. *coli*, with a physics-based model. It is well known that the cytoplasm of prokaryotes such as *E*. *coli* is highly crowded with a large number of macromolecules including protein, DNA and RNA, the concentrations of which are estimated to be 200–320 mg/ml, 11–18 mg/ml and 75–120 mg/ml, respectively [14], [15]. Moreover, these macromolecules occupy 20–40% of total volume of the cell [15]–[17]. Hence, the problem of modeling a cell is one of modeling a concentrated solution of biomolecules. This high macromolecular concentration induces large excluded volume effects that modify the macromolecular properties inside the cytoplasmic environment [15], [18]–[26]. Moreover, the variety of biomolecules present in a cell makes it a highly heterogeneous system.

In the past few years, various experimental techniques have been employed to study the behaviors of macromolecules in the cellular environment. Experimental methods include single particle tracking (SPT) which involves selective labeling of proteins with fluorophores like green fluorescent protein (GFP) and tracking their motion using suitable camera detectors [27]. Other most widely used techniques to study mobility of macromolecules inside the cytoplasm are fluorescence recovery after photobleaching (FRAP) and fluorescence correlation spectroscopy (FCS) [27], [28]. These techniques, however, are limited in that it is difficult to obtain the dynamics of many particles at the same time. On the other hand, computer simulation, in principle, can monitor the dynamics of all particles of a system simultaneously.

There have been several reports where simulations of concentrated solutions of proteins were performed [29]–[32]. Although these are not meant to simulate a full cell, the basic physics, such as excluded volume and hydrodynamic interactions (HI), remain similar. Volume exclusion for a molecule refers to the unavailability of a portion of the space because of the presence of other molecules. HI can be defined as follows: in a fluid, any fluctuating particle induces a velocity field in the solvent, which affects the motion of other particles present in the fluid. This interaction among particles mediated by a fluid is termed HI. As far as modeling of the interior of a cell from a molecular perspective is concerned, only a few studies have been reported. The group of Martin Field, in their pioneering work, modeled a collection of proteins, t-RNAs and ribosomes with spheres [6]. They included both short-ranged Lennard-Jones and long-ranged electrostatic interactions in their model. They have considered both structural properties like structure factors, pair-correlation functions and dynamic properties like diffusion coefficient (D). In a seminal work, the Elcock group modeled the bacterial cytoplasm using all atom models for the 50 most abundant proteins of *E*. *coli* [7]. The interactions between the proteins were treated with electrostatics and a Lennard-Jones potential. In this initial study, the Elcock group did not include HI between the proteins. Their initial model was not able to reproduce the experimental D of GFP, presumably because of neglect of HI; with modifications to the Van der Waals' parameters, the model was able to reproduce the experimental D of GFP.

Ando et al. [8] have carefully treated the HI using Stokesian dynamics [33] in their modeling of *E*. *coli*. This model was successful in reproducing the D of GFP in a simulated cellular environment. It also determined various factors affecting the diffusivity of proteins in a crowded cytoplasm, such as the size and shape of proteins, and HI. The main limitation of this model is the large computational cost of calculating the HI, as their implementation involves a Cholesky decomposition of the diffusion tensor matrix, which scales roughly as the cube of the number of particles. Using the idea of hydrodynamic screening in a subsequent work, Ando et al. [34] approximated the far-field part of Stokes dynamics [33] by a diagonal matrix, thus screening the long-range HI completely. This approximation reduces the computational scaling to *O(N)*, where *N* is the number of particles. Wang et al [9] developed a coarse-grained model of *E*. *coli* cytoplasm to investigate protein stability inside the crowded environment of a bacterial cell, using a model containing the fifty most abundant macromolecules that together account for 85% by weight of the cytoplasm protein content. Trovato et al. [10] presented a multiscale model to predict mobility of GFP within the *E*. *coli* cytoplasm, combining a coarse-grained model of GFP in which each sphere represents one residue with a mesoscale model of cytoplasm. The model accurately predicted the effect of macromolecular crowding on GFP diffusion, although the friction coefficient of GFP had to be parameterized.

In this work, we consider the most abundant proteins of *E*. *coli* in our cytoplasm model, describing each protein as a flexible unit consisting of a collection of spheres. Another major departure from the previous models is that we accounted for hydrodynamic effects with a simple mean field approximation [35]–[39] based on a dynamic scaling of D of the spheres by their local volume fraction. Our use of a simple HI model allowed us to run multiple independent trajectories to assess the statistical error of the calculated observables. With our model we could accurately estimate the D of GFP inside the cytoplasm of an *E. coli* cell and also calculated D of two other proteins. We have also investigated anomalous diffusion (AD), which has been observed in some experiments [40]–[45], using Fractional Brownian Motion (FBM) analysis. It was found that AD was present although deviation from normal diffusion was small. The underlying physical reasons for the AD in our model were identified by running a series of control simulations in which the different interactions used to describe the cytoplasm were modulated.

## Materials and Methods

### Cytoplasmic model

**Figure 1:**
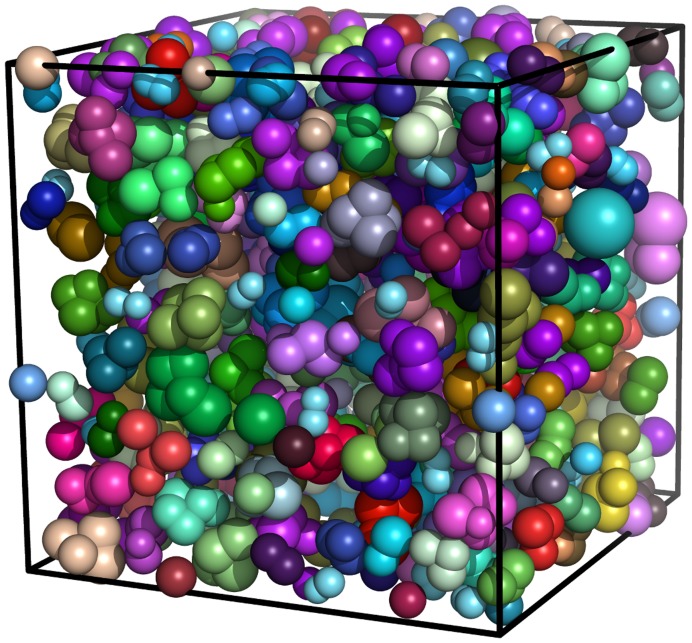
Virtual cytoplasm model in a cubic box of length 406 Å. Each protein is represented by a collection of spheres representing the volume and approximate shape of a particular macromolecule.

The cytoplasm has been built using the most abundant proteins in *E. coli* by taking their structures either from the Protein Data Bank or using homology modeling. The structures have been coarse-grained using *k*-means clustering of the alpha carbon atoms, where the number of clusters is determined by the molecular weight of the protein (1 cluster for <5 kD, 2 clusters for 5–14 kD, 3 clusters for 14–23 kD, 4 clusters for 23–32 kD, and 5 clusters for >32 kD). Each cluster is then represented by a sphere. The radii of the spheres are proportional to the number of residues represented in the cluster, with scaling applied to ensure that the total volume matches that of an all-atom model (taking sphere overlap into account). The relative abundance of each protein present in E. *coli* has been reported previously [46]. Based on the relative abundance of these proteins, the 159 most abundant proteins along with GFP were modeled in a cubic box of length 406 Å. The number of copies of each protein is calculated from their whole-cell abundance. Figure 1 shows this cytoplasmic model of *E. coli* in a cubic box of length 406 Å.

### Energy Model

As described in the preceding section, our model represents not only the size of macromolecules but also, at a low level of resolution, their shape, by using multiple spheres for all but the smallest macromolecules. For the Brownian dynamics simulations, the spheres need to be connected appropriately, and we chose to do so using stiff harmonic potentials. Specifically, the energy potential connecting the spheres together consists "stretching" potentials and "bending" potentials as given below. The stretching potential E_s_ is given by
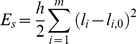
(1)In the above equation 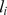 is the *i-*th bond length in a protein, 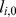 is the equilibrium i-th bond length, 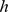 is the stretching force constant (taken as 0.06 kcal/mol/ (Å^2^)) and *m* is the number of such "bonds", which connect every pair of spheres. The bending potential E_b_ is given by
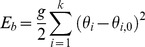
(2)In the above equation g is the bending rigidity constant (taken as 0.006 kcal/mol/ (radian^2^)), 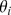
and 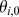 are the i-th bending angle and equilibrium bending angle respectively and 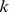
is the total number of angles in the system. The only inter-protein interaction in the model (apart from HI) is a quadratic harmonic potential (E_r_) that was used to disallow overlap of two proteins:
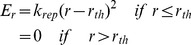
(3)where 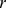
is the distance between two spheres of different proteins and 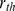
is defined to be the sum of the radii of the same. 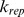 is a parameter used to modulate the repulsive interaction, whose value was taken as 0.1 kcal/mol/Å^2^ unless otherwise mentioned.

### Brownian Dynamics

Brownian dynamics simulations for the cytoplasmic model are performed in a periodic box. The simulation follows the Langevin dynamics according to the following equation 
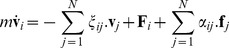
(4)where *N* is the total number of spheres in the system, 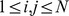, *m* is the mass of the i-th sphere, 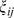
is the friction tensor related to the coefficient 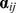
by
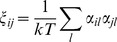
(5)

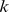
and 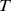
are Boltzmann constant and temperature respectively. 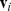
is the velocity of the i-th sphere and 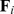 is the sum of forces acting on sphere *i*. The last term on the right hand side of eq. (4) is a random fluctuating force exerted on particle i by the surrounding fluid. 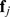
is calculated from a Gaussian random number distribution with 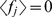 and 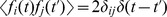 (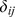
and 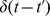
are Kronecker and Dirac delta, respectively and 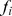
is the scalar component of 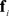).

When the momentum relaxation of the system is much faster than the position relaxation, then following the Ermack-McCammon approach [47], the Langevin equation can be transformed to the following Brownian dynamics simulation protocol.
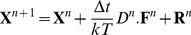
(6)In the above equation 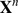
is the position of the centre of the spheres at step n, *D^n^* is the translational diffusion coefficient (which is, in general, a tensor; however, in the present work it is taken as a scalar), 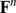
is the force vector and 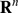 is the random displacement, all are at step n. 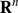
is generated from a Gaussian distribution with zero mean and variance 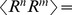 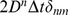, where 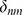
is the Kronecker delta and 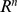 is the scalar component of 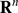.

The translational diffusion coefficient *D* of each sphere is calculated by rescaling the diffusion coefficient at infinite dilution with the local volume fraction (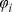) occupied by the sphere [35]–[37]. The volume fraction is calculated in the following manner.

**Figure 2:**
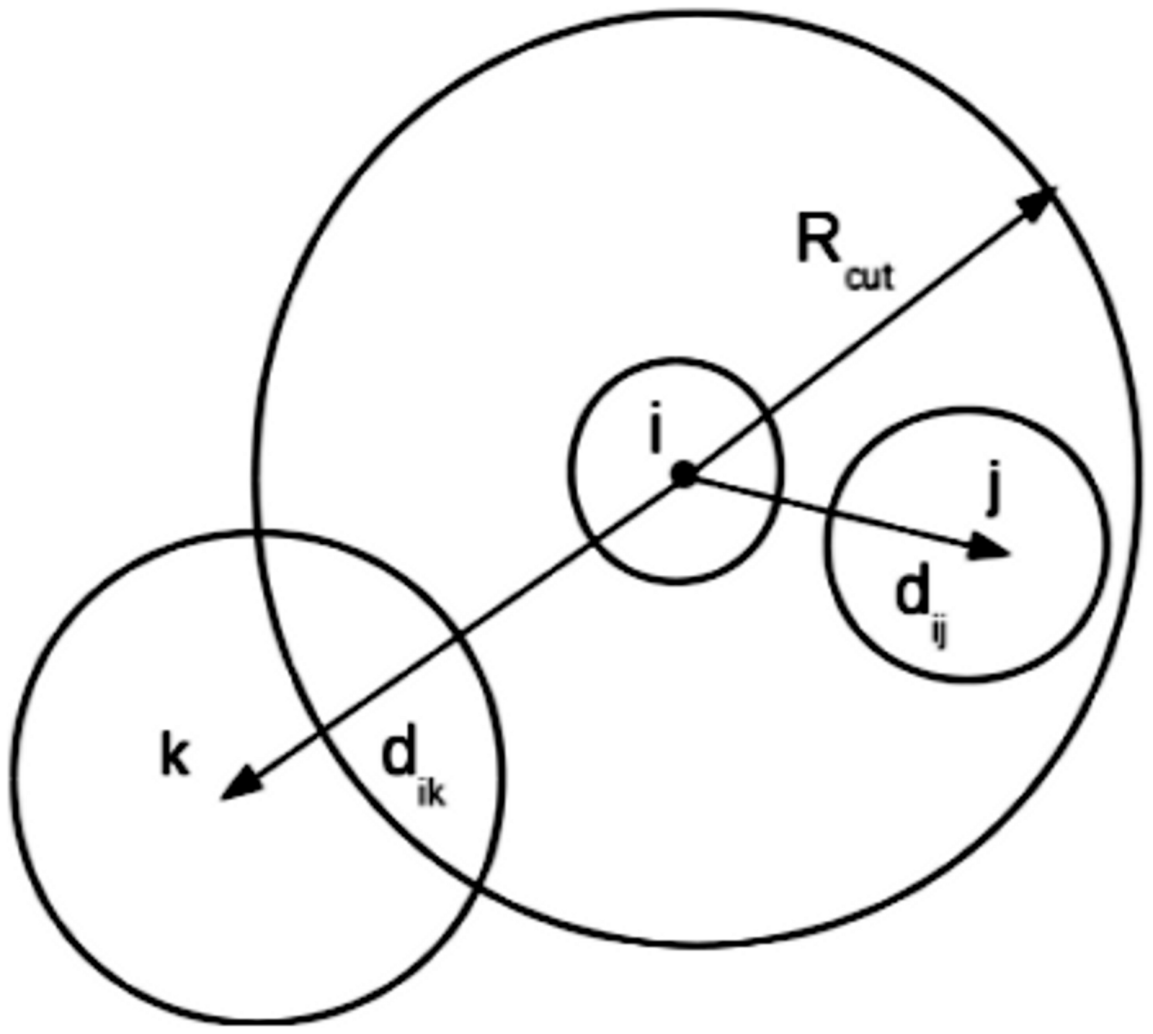
Model for the calculation of local volume fraction for the sphere i. In this model, R_cut_ is 4 times radius of central sphere i, and j and k are spheres that lie completely inside and partially inside R_cut_, respectively.

The local volume 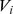 is defined as the volume of a sphere having radius *R_cut_*, where *R_cut_* is four times the radius of the sphere *i*. The local volume fraction (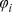) for the sphere *i* is defined as the volume of all spheres which lie within *R_cut_* divided by the local volume 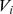.
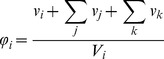
(7)where 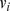 is the volume of the i^th^ sphere. 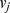
and 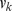 are the volumes of spheres lying completely and partially inside the volume 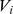, respectively. Figure 2 shows the local volume for the sphere *i*. In the figure, sphere *j* lies completely inside 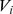, whereas sphere *k* lies partially inside 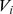.

The short time translational diffusion constant can be defined following the work of Tokuyama et al. [38], where they investigated the dynamics of concentrated hard-sphere suspension, as 
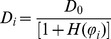
(8)where 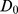
is the translational diffusion coefficient at infinite dilution, 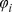
is the volume fraction for sphere *i*, *H*(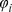) is a scalar function defined as 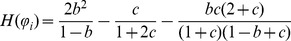
(9)where b = [(9/8) 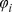]^1/2^ and c = (11/16) 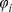.

### Simulation Details

Simulations were performed in a cubic box of length 406 Å with periodic boundary conditions, which keeps the number density inside the box constant. The system was simulated with a time step of 0.01 ns. We generated five different trajectories of 1 ms in length from different random starting configurations. Later, to understand the effect of HI and repulsive interactions on the AD, several simulations were run, in which the repulsive potential and/or HI were turned off. Details of these simulations are given in the next section. A movie showing a part of one trajectory is shown in the Movie S1.

## Results and Discussion

Although any quantity that is a function of sphere coordinates can be calculated from the simulation trajectory, our main emphasis in this work is diffusion. For this purpose, the diffusion coefficient of a "virtual" GFP was calculated and compared to the experimentally measured diffusion coefficient. GFP was modeled as a collection of spheres as done for other proteins and only one GFP was used in our simulation. We also looked for evidence of AD, as it is known that the crowded environment inside a cell can give rise to AD.

### Comparison of calculated and experimental translational diffusion coefficients

**Figure 3:**
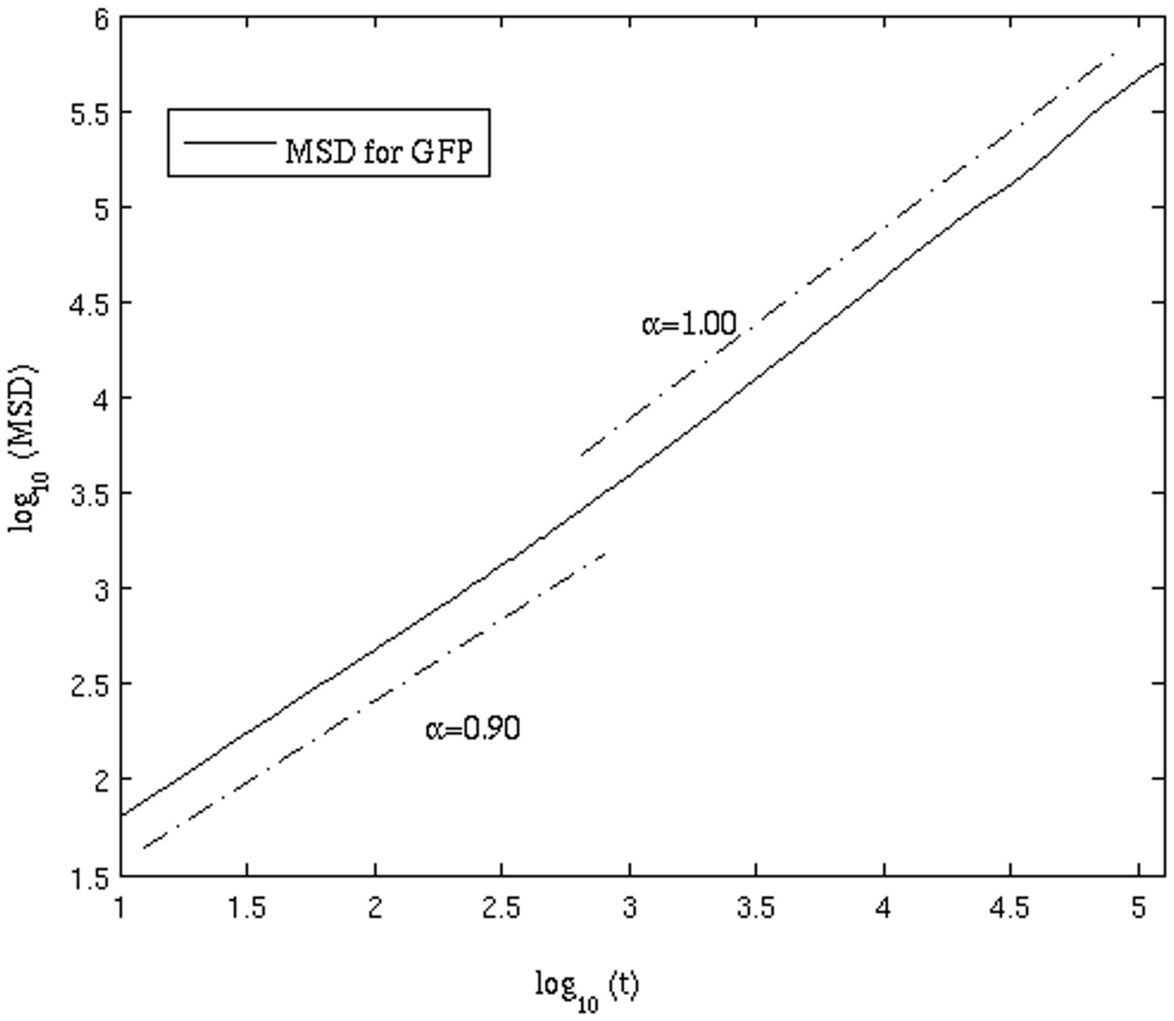
Scaling of the mean-squared displacement as a function of time.

The position of each sphere was recorded at regular intervals of 10 ns. The mean square displacement (MSD) of the centre of mass (COM) of a protein was calculated (shown in the right hand side of eq. 10) and averaged over different time origins. From the MSD, the diffusion coefficient (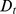) was calculated according to 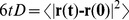
(10)where *t* is the time difference, **r**(t) and **r**(0) are the COM position vectors of the system at time *t* and 0 respectively. For normal diffusive motion, the MSD should show a linear dependence on time, as can be seen from equation (10). To obtain a clearer picture of how MSD actually varied with time difference in our simulation, a log-log plot of MSD versus time is shown in Figure 3. Linear least squares regression was performed on varying regions of the plot to determine the dependence of MSD with time. From Figure 3, we see that at short time scales (approximately 10 ns<t<1 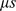) the MSD does not show linear scaling with time (MSD 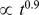). Normal diffusion is observed in the time range from 1 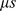
<t<100 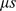
as in this region MSD 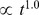. Beyond that the data was not reliable because of inadequate statistics (data not shown).

For the calculation of *D*, a block averaging method is used. In this study, each trajectory has been divided into two blocks, each of length 400 µs (first 200 µs simulation data was discarded from the analysis). We calculated the MSD for each block and plotted it against the time difference. Linear least squares regression was done in the region of the plot showing linear scaling with time (MSD 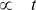). From the linear region in the log-log plot, the intercept value yields the value of the diffusion coefficient, *D*.

The diffusion coefficient for GFP found in our study, 6.51±0.47 µm^2^/s, was close to the reported experimental values of 7.7±2.5 µm^2^/s [48] and 6.1±2.4 µm^2^/s in the cytoplasm of *E. coli* [49]. The calculated value of the diffusion coefficient of GFP in the cytoplasm is about eleven times lower than its value in dilute solution, which is 87 µm^2^/s [50], [51]. The reduction in the diffusion coefficient inside the cytoplasmic environment can be mainly attributed to the volume exclusion and HI [8].

**Figure 4:**
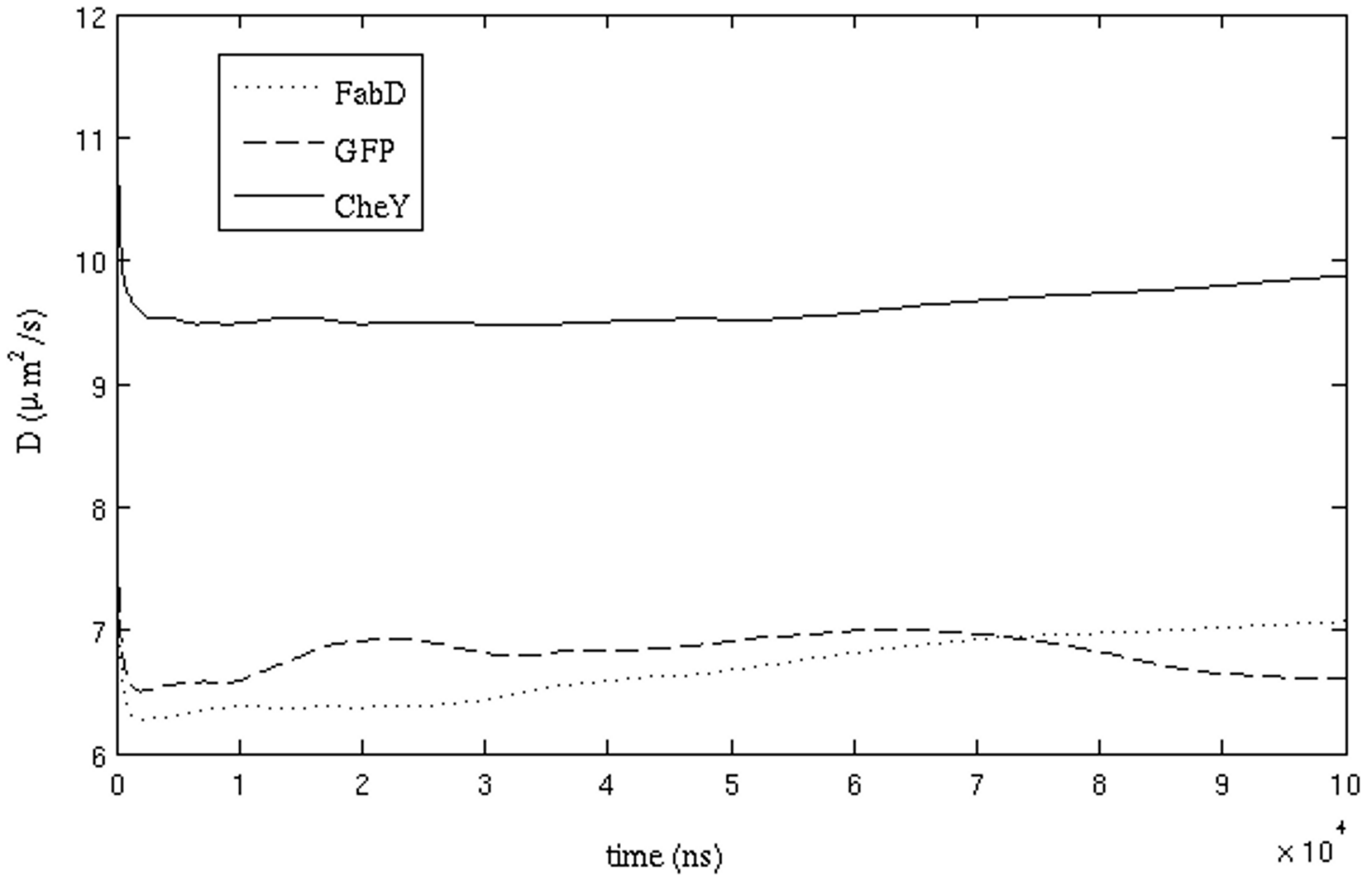
Diffusion coefficient values of the particles corresponding to GFP, CheY and FabD plotted against time.

We have also calculated the diffusion coefficients of two other proteins, namely the chemotaxis protein CheY and malonyl CoA-acyl carrier protein transacylase (FabD). The simulated value of the diffusion coefficient of CheY (9.42±1.12 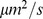) is found to be close to the value of 10.0 (±1.3) 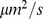
given by Lipkow et al. [52] in their model of *E. coli*. For both CheY and FabD (whose D value is 6.26±0.66 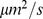), as with GFP, the diffusion constant in the simulated cytoplasm was roughly a factor of ten lower than that in dilute solution. The molecular weight of FabD (∼32 kD) is the largest among the three proteins, and as would be expected, its calculated diffusion coefficient is lower than that of other two proteins. CheY has the lowest molecular weight (∼14 kD) among the three and has the highest value of D. Table 1 shows the values of D from individual trajectories for the three proteins. Figure 4 shows the time-variation of *D* for GFP, Chey and FabD. At longer time scales, the *D* values of CheY and FabD essentially converge and are time-independent. For GFP, *D* values show about 10% fluctuation from its mean value of 6.51
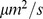
_._

**Table 1:** Diffusion coefficient values for three proteins with and without considering hydrodynamic interactions (HI). SD represents standard deviation.

Trajectory	Diffusion coefficient (±SD) in units of µm^2^/s					
	GFP		CheY		FabD	
	With HI	Without HI	With HI	Without HI	With HI	Without HI
**1**	6.32 (0.23)	16.26 (1.44)	9.39 (0.83)	25.20 (3.72)	5.96 (0.37)	13.76 (1.93)
**2**	6.55 (0.10)	16.30 (0.13)	9.45 (0.57)	22.84 (1.71)	6.16 (0.21)	14.70 (0.78)
**3**	6.78 (0.08)	16.08 (0.74)	8.94 (1.32)	23.58 (1.08)	6.48 (0.18)	14.88 (0.76)
**4**	6.44 (0.47)		9.71 (0.30)		6.32 (0.66)	
**5**	6.46 (0.44)		9.60 (1.12)		6.39 (0.17)	

One issue is how the D values depend on the choice of the repulsive force constant. To check that we have run simulations with the value of force constant as 1.0 kcal/mol/Å^2^. The D values were found to reduce by about 6–10% (D values are 6.1±0.22, 8.8±0.64, and 5.6±0.62 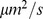 for GFP, CheY and FabD, respectively) than the values obtained from simulations with force constant of 0.1 kcal/mol/Å^2^. This is because higher repulsion reduces the mobility of the proteins.

### Role of Hydrodynamic Interactions

Hydrodynamic interactions (HI) are known to play a major role in the diffusivity of proteins in crowded environments. To examine the effect of HI on the calculated diffusion coefficient, control simulations were performed without HI. Table 1 compares the diffusion coefficients for the three proteins with and without HI. In all cases, neglecting HI increases the observed diffusion coefficients by a factor of 2–3. As expected, HI retards the motion of proteins in the crowded environment.

### Analysis of anomalous diffusion

There have been several investigations of anomalous diffusion (AD) in crowded environments [40], [41], [44], [53]. One mechanism for AD is that over certain time scale, the successive steps a particle takes are correlated, unlike in normal diffusion, where steps are uncorrelated. There are several models such as obstructed diffusion (OD), fractional Brownian motion (FBM), and continuous time random walk (CTRW) to explain AD. For instance, in the FBM, the probability distribution of the process is Gaussian but it is non-Markovian. On the other hand, in the CTRW model, it is both non-Gaussian and non-Markovian. It is also reported in the literature that the same data can be explained by different models, suggesting that a definitive mechanistic explanation may be elusive [54]. Although AD has widely been observed as a nonlinear growth of MSD, often the properties of the associated propagators are hard to find. Weiss et al. [54] have grouped the propagators for subdiffusive motion into two categories based on a stationary increment (i.e. the increment for a time slice depends only on the time difference) or a non-stationary increment of steps. A stationary increment gives rise to FBM while a non-stationary increment gives rise to CTRW which, in contrast to FBM and OD, shows weak ergodicity breaking. Weiss et al. utilized fluorescence correlation spectroscopy (FCS) to determine the propagator responsible for crowding-induced subdiffusion, and found that subdiffusion in a crowded environment is most consistent with a stochastic process with stationary increments, characteristic of FBM [55].

In the current work, we have performed simulations where the random force term is uncorrelated in time. However, even in that case AD may occur due to the crowded nature of the system. The crowding can be mapped implicitly to a simpler system using a memory-dependent random force in the Langevin equation as done by Weber et al [56]. *We have investigated whether our trajectories can be represented as a memory-dependent random force acting on GFP and whether they follow FBM*. The two main conditions for FBM are that (a) the probability distribution of displacement should be Gaussian and (b) there should be negative value for the displacement auto-correlation function (shown below) for AD [54]:
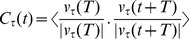
(11)where, 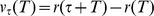
is the increment vector of position r between time T and 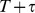
for the particle under consideration. For normal diffusion 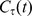
will tend to zero for 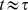. However, for FBM, this will lead to a negative value of 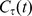 [55].

We have found that the probability distribution of displacement in our simulations is approximately Gaussian (shown in Figure S1) and it indeed shows negative values of the correlation function. The (small) deviation from Gaussian distribution can be from several reasons including lack of enough statistics for this highly concentrated system. The interpretation of the analysis discussed later should be taken with this caveat. Moreover, no attempt was made to investigate other models of diffusion in this work. We have used well defined correlation function in FBM, which gives signature of AD. This analysis is similar in spirit to the works of Weiss et al., where FBM was used to find signature of AD from their experimental trajectories [54].

**Figure 5:**
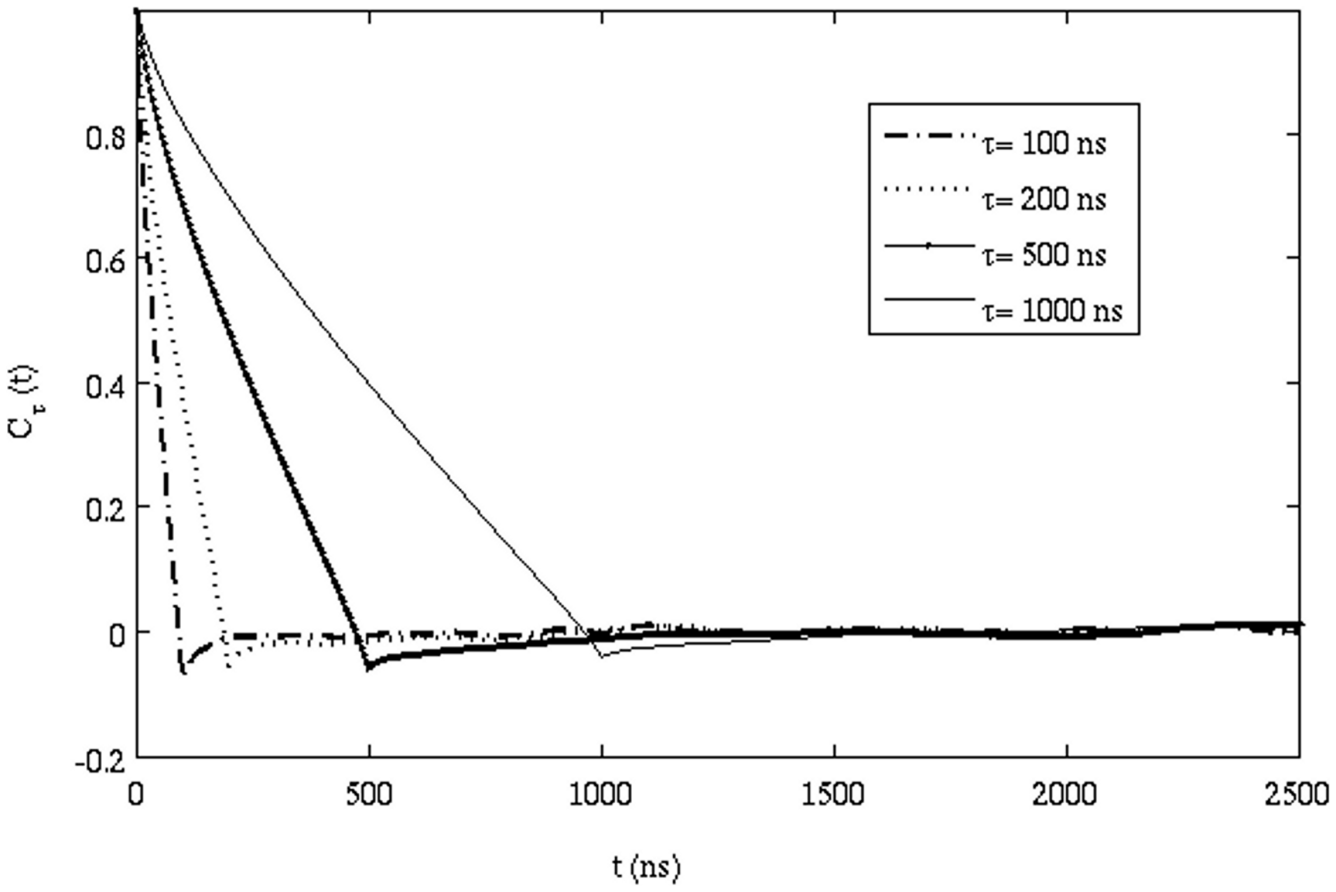
Correlation function of successive displacements (eq. 11) for different time lags (see text) ranging from 100 ns to 1000 ns.

**Figure 6:**
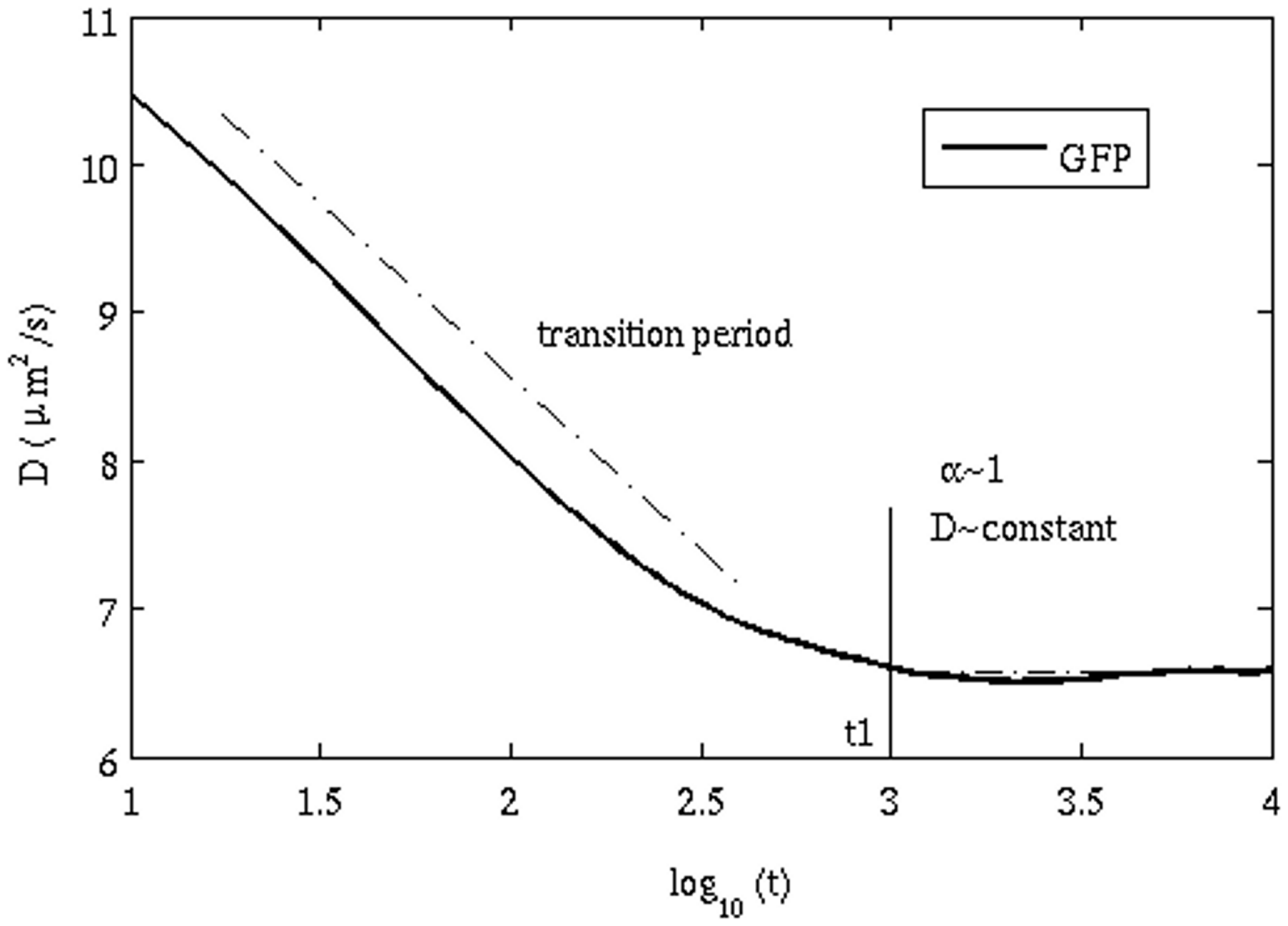
Anomalous subdiffusion occurring over short time regimes, tending to normal Brownian motion at larger time differences. Diffusion is subdiffusive anomalous for t<3 on the log scale. After this time, the diffusion is approximately "normal" Brownian motion.

We have calculated 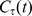
for the displacement of the GFP protein for 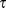 = 100, 200, 500 and 1000 ns. To improve statistics, we ran an additional 1 ms simulation where we have saved coordinates every 1.0 ns. Figure 5 shows 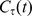 for various 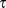. It is clear that for 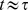, 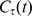 is less than zero when 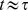, characteristic of AD, and gradually decays to zero as t increases, characteristic of a Markovian random walk at later time intervals. The minimum value of 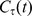 and the exponent alpha (MSD 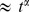) is related by 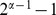 [57]. The exponent 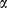 is found to be approximately 0.90, suggesting that the effect of AD is small. The onset of AD and its gradual change to normal diffusion can also be seen from the time-dependent diffusion coefficient (Figure 6). The D value remains independent of time only after a certain time lag t_1_ (which corresponds to ∼1 µs); before that D decreases as the time lag increases.

### Effects of hydrodynamic and repulsive interactions and mobile species on anomalous diffusion

To investigate the underlying physical basis for AD, we performed additional simulations to observe the effects of removing HI, removing the repulsive interactions, and eliminating the mobility of the other protein species.

**Figure 7:**
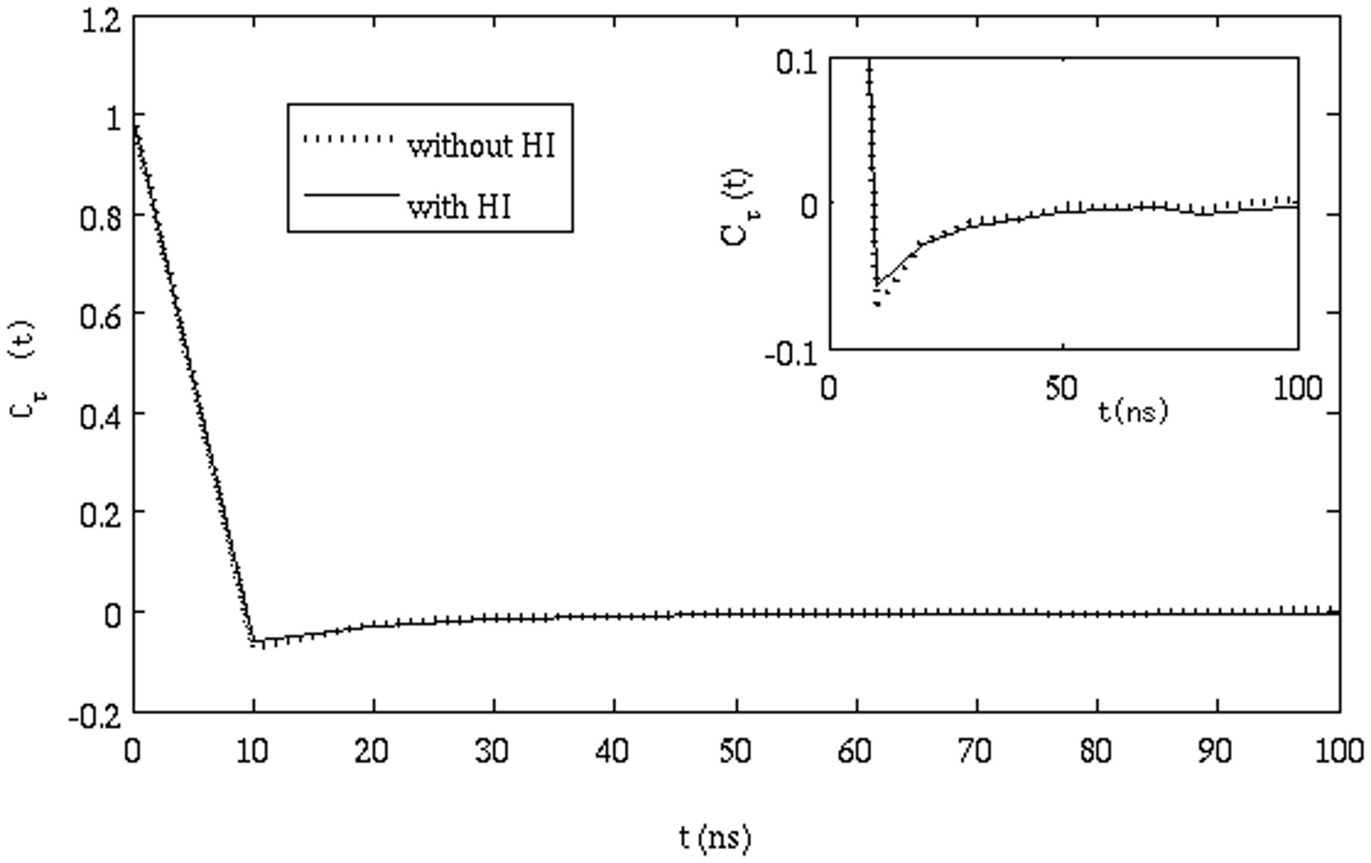
Correlation function of successive displacements (eq. 11) for simulation with and without hydrodynamic interactions.

**Figure 8:**
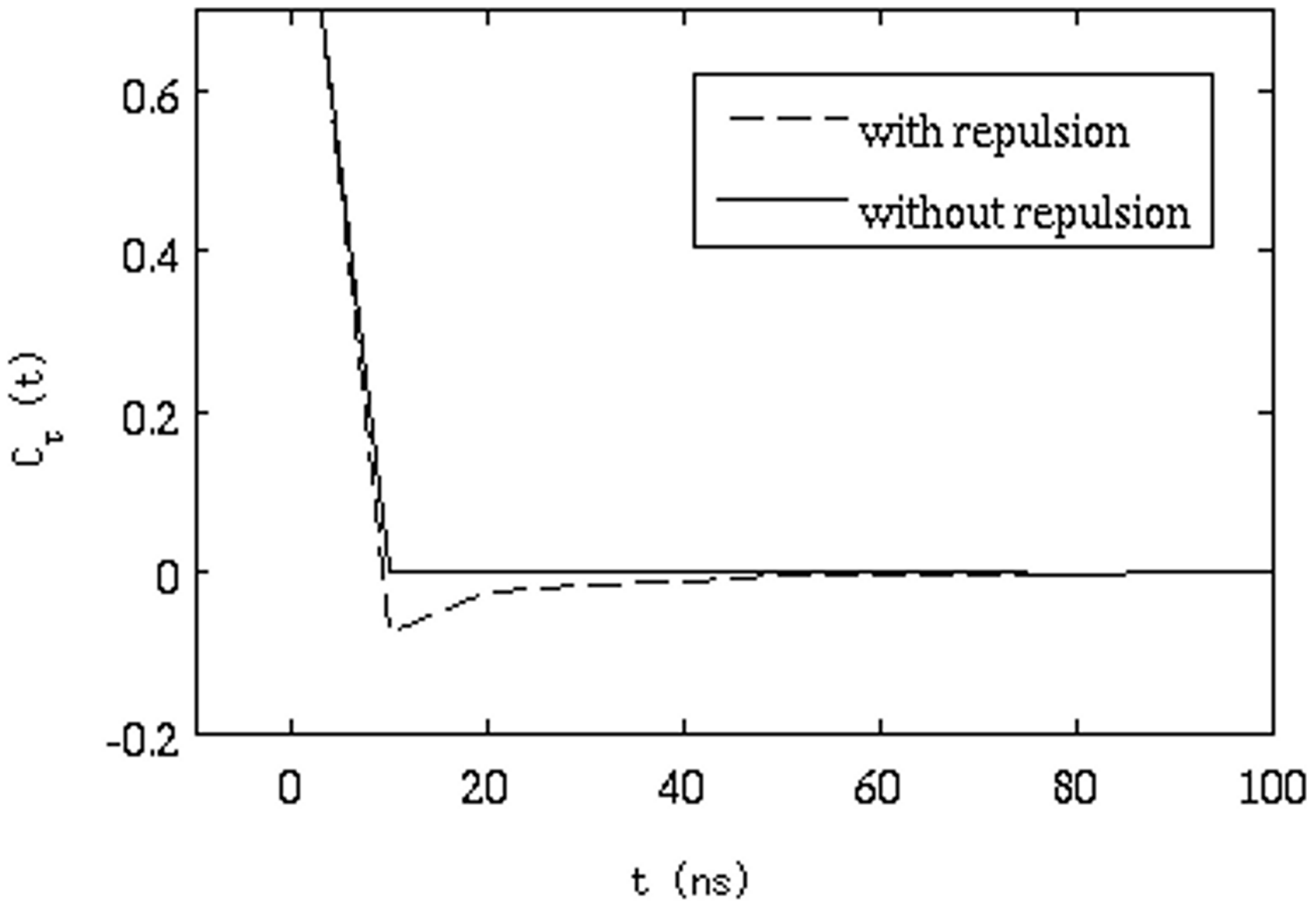
Correlation function of successive displacements (eq. 11) with and without repulsive interactions (both are without hydrodynamic interactions).

**Figure 9:**
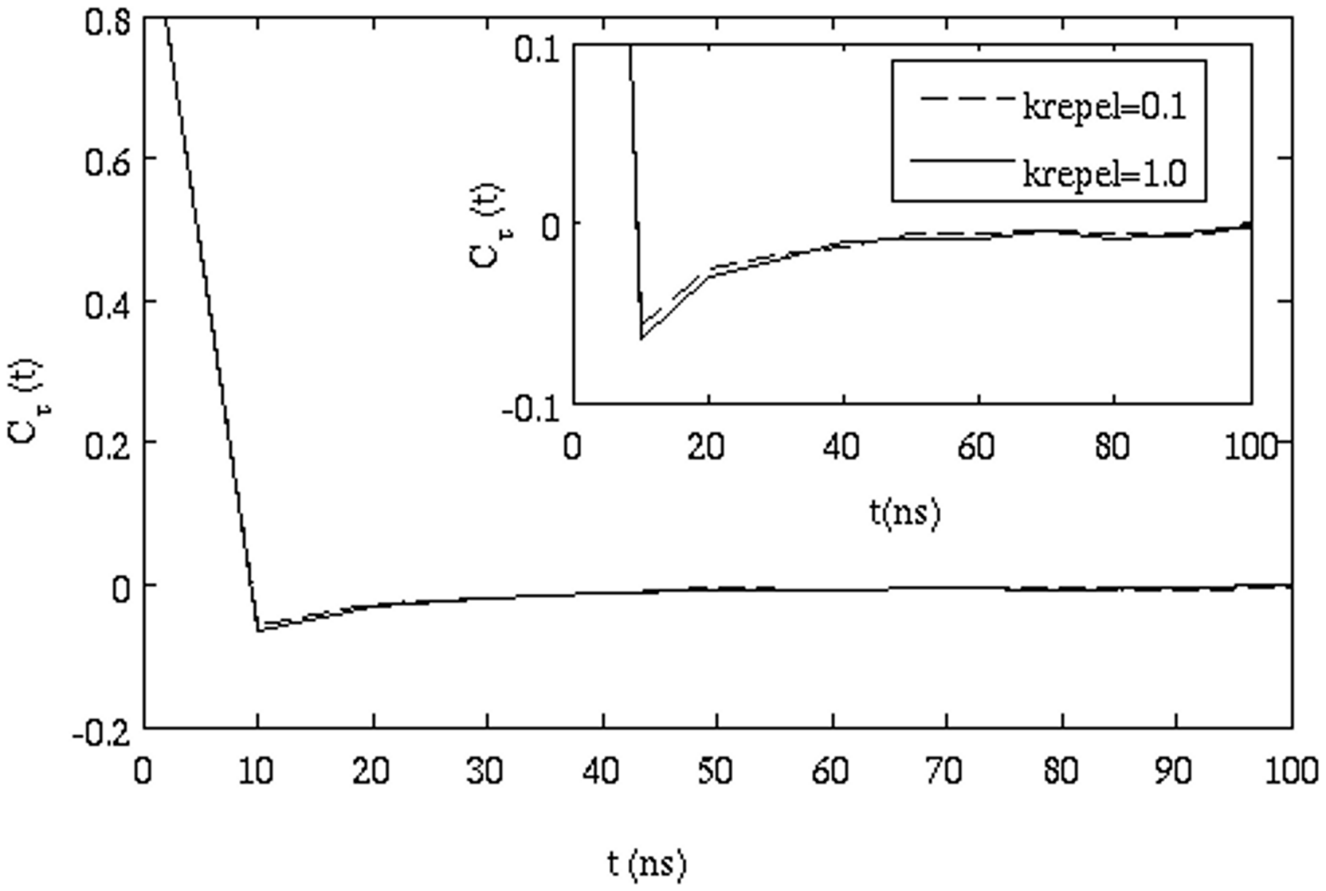
Correlation function of successive displacements (eq. 11) for two different repulsive force constants (both are with hydrodynamic interactions). Inset shows the same figure for a smaller range of y-axis.

**Figure 10:**
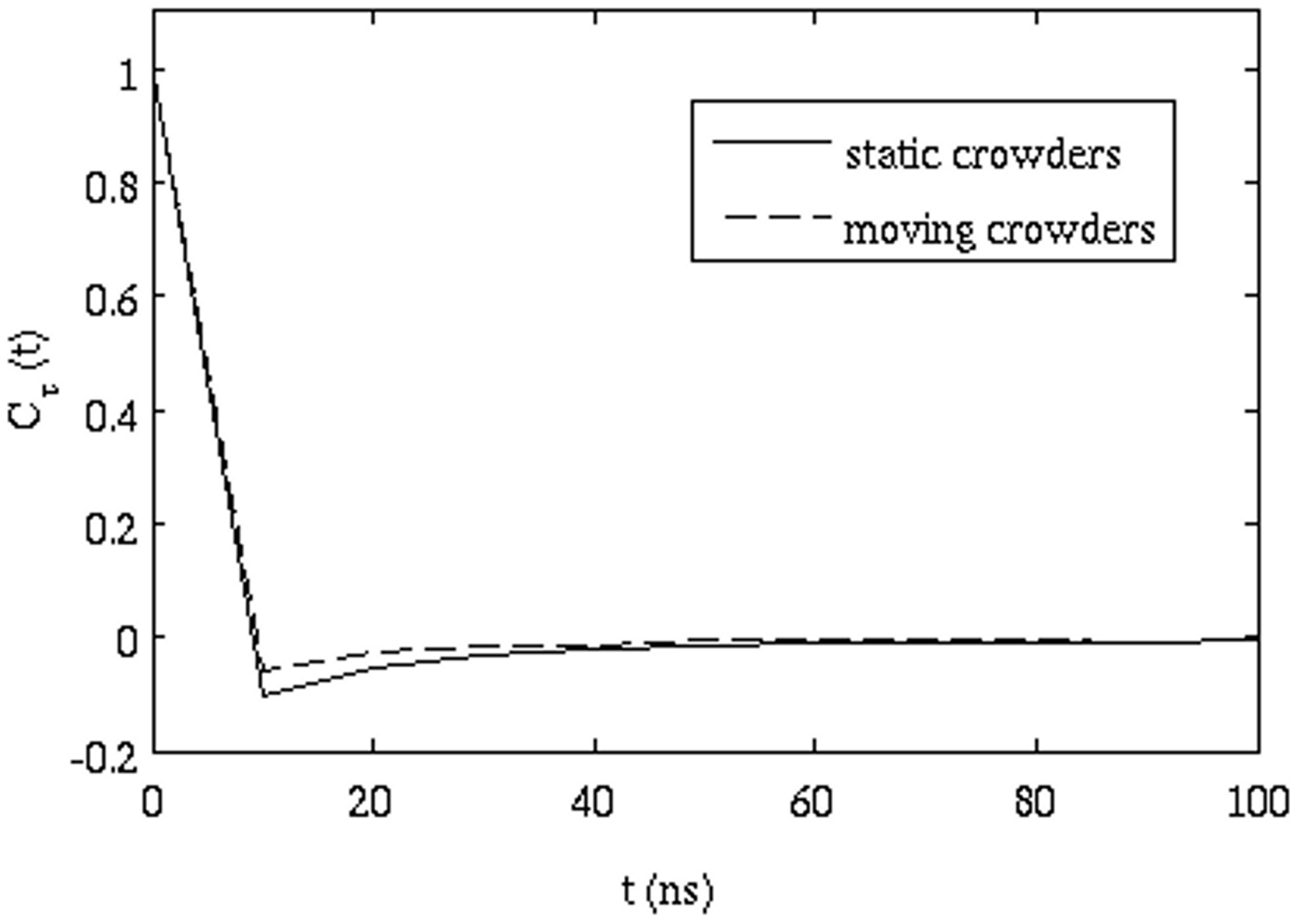
Correlation function of successive displacements (eq. 11) showing the effect of stationary and mobile crowders.

1. **Effect of HI**: To understand the effect of HI, simulations were run without considering HI. The autocorrelation function shown in eq. (11) was calculated from this simulation and compared to the case where HI was present. Figure 7 shows the plot of 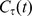
with t. It can be seen from the figure that the minimum value of 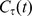 for the simulation without HI is only slightly less than that of 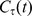
calculated from simulations with HI. Although, the difference is small, it was found to be present by comparing the results for four trajectories in each case.
2. **Effect of excluded volume**: As a control, we performed two 1 ms simulations without HI in the presence and absence of the repulsive term (simulations with HI and without repulsive interaction was not stable due to very large volume fractions during HI calculation). 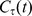
in figure 8 clearly shows that when the repulsion between the proteins is absent, there is no AD, as would be expected when excluded volume effects are eliminated (each 'molecule' effectively diffuses independently). To investigate the sensitivity of the results to our choice of parameters for the repulsive term, we increased the force constant of the repulsive parameter from 0.1 to 1.0 kcal/mol/Å^2^ and performed two set of simulations for each repulsive parameter with HI. Figure 9 shows that the minimum value of 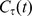 is slightly lower for the higher repulsive force constant, suggesting that higher repulsion between proteins may increase the correlation between the steps that each protein takes in the simulation. However, the difference of 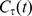 is too small to make any definitive conclusion.
3. **Effect of mobility of proteins on the anomalous diffusion**: We performed another set of simulations where all proteins except for GFP were kept fixed. In this case, the minimum value of 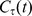 attains a value lower than that calculated from mobile crowders. Figure 10 shows that the presence of fixed obstacles makes the diffusion more anomalous as compared to the mobile crowders. From previous reports [58], it was argued that immobile obstacles are required for AD and that diffusion is approximately "normal" in the presence of mobile obstacles. This is in accord with our findings.

Previous studies suggested that a plausible cause of AD inside the cytoplasm was the heterogeneity of the cytoplasmic environment and the various interactions among the macromolecules, such as excluded volume effects and hydrodynamic interactions [40], [41]. Within our simplified model, which includes only HI and excluded volume interactions between the molecules, we studied how altering these forces affects the anomalous subdiffusivity inside the simulated cellular environment. Our results indicate that the excluded volume effect is one of the main causes of AD. As we can reproduce the experimental diffusion coefficient of GFP in realistic coarse-grained model of the bacterial cytoplasm, we suggest that the excluded volume effect might be one of the main causes of AD in *E*. *coli*.

When we switched off the repulsive interactions in the absence of HI, no AD was observed, suggesting that excluded volume effects promote subdiffusion. On the other hand, hydrodynamic interactions increase the diffusion exponent (albeit by a small amount), thus decreasing the anomalous subdiffusion. As the proteins are (almost) impenetrable and the treatment of HI was to mimic an incompressible fluid, HI is repulsive for particles approaching each other and attractive for particles moving away from each other [8]. For simulations with excluded volume, when particles move apart due to repulsion, they are "attracted" to each other due to HI. Note that no electrostatic interactions were considered in the current work. Although it is likely that long-range electrostatic interactions will be screened by ions present in the cytoplasm, these interactions would still affect interactions between nearby proteins, which may in turn affect transport properties discussed in this work. However, the effect of electrostatics is likely to be minor in determining diffusion coefficient, since our computed results are close to experimental ones even without electrostatics. We reiterate that the analysis of AD is done based on the approximate connection between simulated trajectories and FBM.

## Conclusions

In this work, we developed a simple model of the *E. coli* cytoplasm and studied the diffusion of different proteins in the model cytoplasm. With the only inter-molecular interactions being repulsive interactions and hydrodynamic interactions, our model can calculate diffusion coefficients for GFP that agree well with experiments. Additionally, we used FBM to investigate the AD we observed, and found that that repulsive interaction between proteins is the largest contributor to AD in our model. It is likely that this model can be further employed to study various biological phenomena, such as binding of transcription factors to DNA, the role of scaffolds in regulatory processes, spatial heterogeneity in *E. coli*, and the localization of proteins in a heterogeneous cellular environment.

## Supporting Information

Figure S1 (a) shows the probability distribution of displacement of GFP in 10 ns from our simulations. This is approximately Gaussian. (b) shows the probability distribution of displacement of GFP in 100 ns from our simulations. This is also approximately Gaussian.
(PDF)

Movie S1 **In this movie, a short part of the Brownian dynamics trajectory of the cytoplasm with GFP (shown in green) is shown.**
(MPG)
