# Supplementary material for: A New Coarse-Grained Model for E. coli Cytoplasm: Accurate Calculation of the Diffusion Coefficient of Proteins and Observation of Anomalous Diffusion
Source: PLoS One. 2014 Sep 2;9(9):e106466. doi: 10.1371/journal.pone.0106466 (PMC4152264; doi:10.1371/journal.pone.0106466)

Figure S1(a): Probability distribution function for the displacement of GFP in 10 ns. The green curve in the figure shows the Gaussian fit of the probability distribution function.

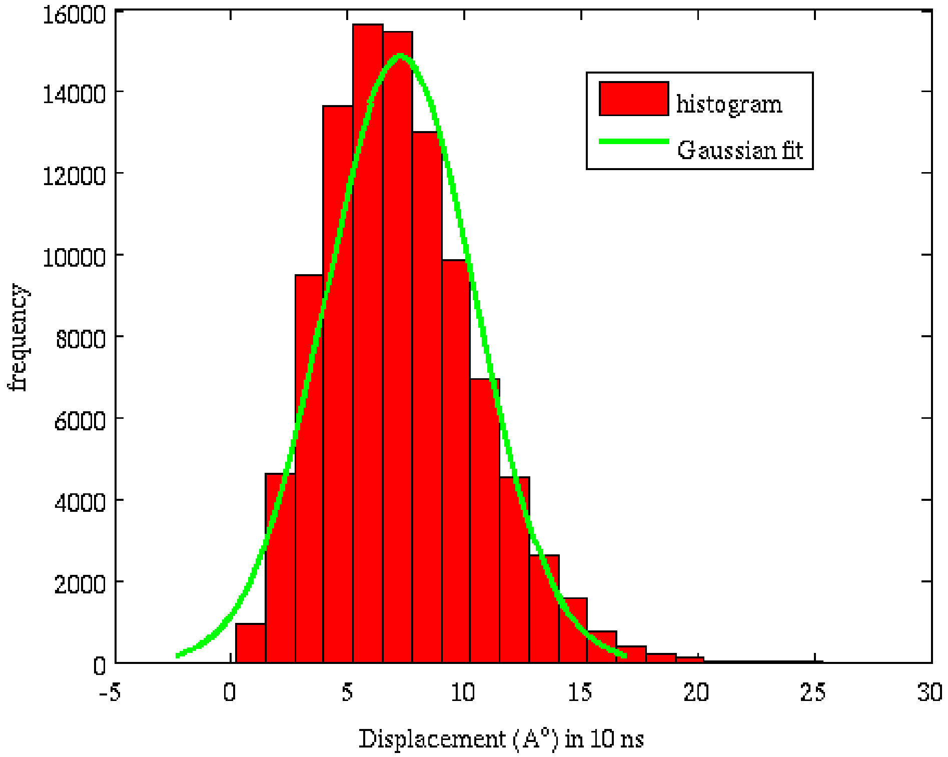

Figure S1(b): Probability distribution function for the displacement of GFP in 100 ns. The green curve in the figure shows the Gaussian fit of the probability distribution function.

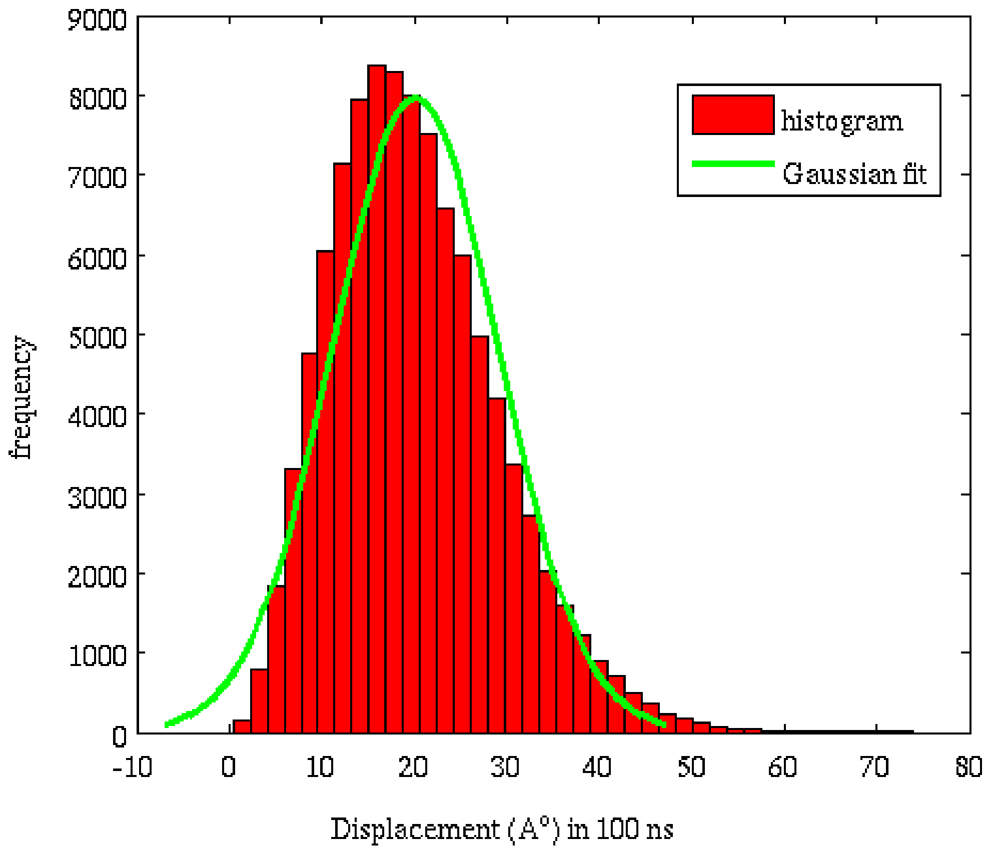

Supplement: Figure S1 — (a) shows the probability distribution of displacement of GFP in 10 ns from our simulations. This is approximately Gaussian. (b) shows the probability distribution of displacement of GFP in 100 ns from our simulations. This is also approximately Gaussian. (PDF) [file pone.0106466.s001.pdf]
